# Selective culling and the potential persistence of lumpy skin disease in cattle herds: insights from a mechanistic transmission model

**DOI:** 10.1186/s13567-026-01828-5

**Published:** 2026-08-29

**Authors:** Clara Delecroix, Gaël Beaunée, Stéphane Bertagnoli, Thibaut Lurier, Brandon Hayes, Sébastien Picault, Guillaume Fournié, Timothée Vergne

**Affiliations:** 1 ENVT, INRAE, IHAP, Université de Toulouse, Toulouse, France; 2 BIOEPAR, Oniris–INRAE, , 44300 Nantes, France; 3 VetAgro Sup, UMR EPIA, INRAE–Université de Lyon, Marcy l’Etoile, France; 4 VetAgro Sup, UMR EPIA, INRAE–Université Clermont Auvergne, Saint Genès Champanelle, France; 5 Department of Pathobiology and Population Sciences, Royal Veterinary College, London, UK

**Keywords:** Epidemiological model, mechanistic model, lumpy skin disease, culling, control strategies

## Abstract

The first epidemic of lumpy skin disease (LSD) in France was detected in June 2025, with a total of 117 outbreaks recorded by the end of the year. This fast-spreading vector-borne disease of cattle prompted the implementation of strict control measures, including total depopulation of affected herds, resulting in the culling of more than 3500 cattle. Over the course of the epidemic, this measure became increasingly unacceptable, leading to major protests. To contribute to this veterinary public health debate, we present a mathematical model, accounting for both cattle and vector populations, that compares within-herd transmission dynamics under selective or total depopulation strategies, and different vector control scenarios. The selective depopulation strategies are modelled based on a bi-daily test-and-cull approach implemented upon detection of the first clinical case in an unvaccinated herd, with imperfect diagnostic tests capable of detecting infection in asymptomatic cattle under different assumptions of test sensitivity and of time from infection to detectability. Our model shows that the selective culling strategy is insufficient to control the spread of the disease in the absence of vector population control, with the entire herd eventually becoming infected. In the best-case scenario with highly sensitive and timely diagnostic tests and an 80% reduction of the vector population, still 75% (95% simulation interval: 26, 100) of the herd becomes infected. Assuming more realistic diagnostic tests (30% sensitivity for detecting subclinical animals within 2–3 days before the onset of infectiousness), the model predicts that 94% of the herd (95% simulation interval: 44, 100) eventually becomes infected, representing only a marginal improvement over a total depopulation strategy.

**Supplementary Information:**

The online version contains supplementary material available at https://doi.org/10.1186/s13567-026-01828-5.

## Introduction

On 29 June 2025, France reported its first outbreak of lumpy skin disease (LSD) on a cattle farm in the Savoie department, likely originating from viral introduction from Sardinia, Italy [1]. This vector-borne viral disease, which primarily affects cattle, spreads rapidly and can lead to substantial production losses and even to mortality rates of up to 10% among symptomatic animals [2]. Because of its impact, LSD has been classified as a category A disease in European Union Animal Health Law, thereby requiring the implementation of strict management measures in affected territories with the aim of achieving rapid virus eradication and recovery of disease-free status [3]. In France, 117 outbreaks were reported by the end of 2025. To reduce the spread of LSD virus (LSDV), veterinary services implemented a strategy combining emergency ring vaccination, movement bans within a 50-km radius of affected farms and the complete depopulation of affected herds. This strategy ultimately led to the culling of more than 3500 cattle.

Over the course of the epidemic, total depopulation of infected herds became increasingly unacceptable to cattle breeders, particularly in areas in the south of the country, despite financial compensations provided by the government. This led to major demonstrations and acts of civil disobedience, with farmers arguing that total depopulation—as compared to selective culling—was excessively distressing, especially as the former was perceived as unnecessary once vaccination programmes had been implemented. This alternative approach, which has been applied in several Eastern European countries with varying effectiveness, relies on repeated PCR tests of nasal swabs, skin and/or blood samples from all cattle on affected farms, with euthanasia restricted to animals testing positive [4]. In France, scientific experts did not support this approach because only a fraction of infected cattle develop clinical signs, and PCR testing of blood and/or skin samples or nasal swabs in asymptomatic animals has imperfect sensitivity. The sensitivity of diagnostic tests for asymptomatic animals is known to be low, but there has been little agreement on precise estimates [5, 6]. New tests relying on cytokines are under development and could improve the performance of such tests [7]. Undetected asymptomatic animals may still contribute to viral transmission by contaminating blood-feeding arthropods [8], potentially allowing the virus to persist within affected herds.

LSD is primarily transmitted mechanically by vectors. Some cases of direct transmission have been reported [9] but vector-borne transmission constitutes the main transmission route [10]. Several vectors have been reported to be able to transmit LSD, such as the stable fly *Stomoxys calcitrans* and the mosquitoes *Aedes aegypti* or *Culex quinquefasciatus*.* Stomoxys* biting flies, due to their higher vector competence, high abundance around livestock and aggressive biting behaviour [11], are considered to be the main vector in France. Its life-cycle is completed in decaying organic matter, such as the manure and moist hay found in close vicinity of cattle on which they feed multiple times a day, with a tendency to move between animals. *Stomoxys calcitrans* populations peak during the warmer months [12, 13] and are considered to be the most abundant in France between June and October [14]. They are relatively sedentary insects, rarely travelling more than 150–300 m away from cattle farms where they can reproduce and feed, although some long-distance movements have been recorded [15]. Due to its vector-borne transmission, the spread and persistence of LSD within a herd depend on an intricate mix of factors, and comparing the efficacy of management strategies is not straightforward.

In this context, mathematical models of LSDV transmission could be extremely valuable. Such models allow in silico simulations of LSDV spread within herds under different management scenarios and enable comparison of viral persistence metrics across these scenarios [16]. However, to provide robust scientific guidance in a highly sensitive political context, models must account for asymptomatic infectious individuals and imperfect diagnostic methods. Through explicitly representing these mechanisms, the outcomes of alternative control strategies can be assessed under the conditions in which they are currently being applied.

Here, we present a within-herd LSDV transmission model designed to capture the key biological features of viral dynamics, including vector-borne transmission, the ability to detect the virus several days before the onset of clinical signs, the possibility for infected animals to remain asymptomatic yet infectious and differences in the duration of infectiousness between symptomatic and asymptomatic individuals. Building on a biologically grounded framework informed by current knowledge of the infection course and disease epidemiology, we explicitly simulated test-and-remove strategies to assess the impact of selective culling under varying diagnostic conditions. Using this approach, we aimed to explore how different combinations of test performance influence viral persistence within a typical cattle herd operating under a selective culling strategy.

## Materials and methods

### Transmission model formulation

**Figure 1:**
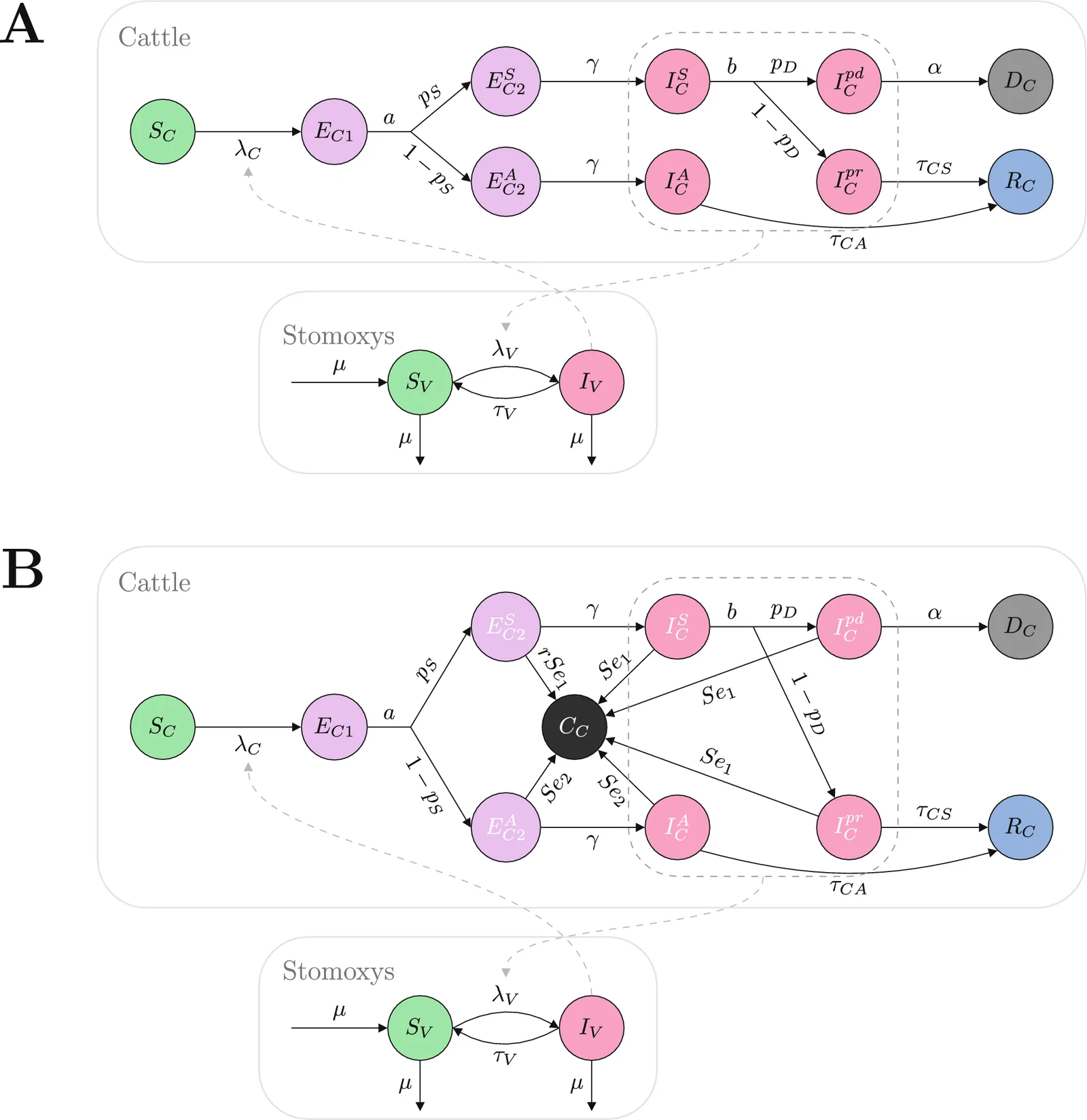
**Compartmental model for LSD within-farm transmission**. The cattle population is subdivided into susceptible (S_C_), latent (E_C1_, ${E}_{C2}^{A}$ and ${E}_{C2}^{S}$), infectious (${I}_{C}^{A}$, ${I}_{C}^{S}$ ${I}_{C}^{pd}$, ${I}_{C}^{pr}$), recovered ®_C_), dead (${D}_{C}$) and culled (${C}_{C}$) cattle. The* Stomoxys* population is subdivided into uncontaminated (S_V_) and contaminated (I_V_).** A** Model without intervention strategies, used until the first detection of an infectious individual.** B** Model with intervention strategies.

We used a Ross-Macdonald-type compartmental model, a widely used framework for vector-borne diseases [17], to represent virus transmission dynamics between cattle and vectors (Figure 1A). The model considers a cattle population of 100 cows (sensitivity analyses are provided in Additional file 3) and a vector population of *Stomoxys calcitrans* with 50 flies per cow, representing a moderate infestation of the farm [11, 18]. We assumed that cattle could become infected only through the bite of an infectious vector and that vectors could become infected only through a bite on an infectious cow. Direct transmission between cows was not considered as little transmission has been reported through that route [9, 10, 19].

The cattle population is subdivided into ten compartments. Upon infection, susceptible cattle (*S*_*C*_) begin their two-stage latent period (compartments *E*_*C1*_ and *E*_*C2*_). After the first stage of the latent period (*E*_*C1*_, lasting $\frac{1}{a}$ days), infected cattle either follow a symptomatic route (with probability *p*_*S*_), leading to the eventual expression of clinical signs, or an asymptomatic route (with probability *1 – p*_*S*_), never displaying clinical signs. Cattle that will remain asymptomatic complete their latent period in ${E}_{C2}^{A}$ after a duration of $\frac{1}{\gamma }$ days, before becoming infectious (${I}_{C}^{A}$) for a duration of $\frac{1}{{\tau}_{CA}}$ days and later recover (*R*_*C*_). Cattle that follow the symptomatic route complete their latent period in ${E}_{C2}^{S}$ after a duration of $\frac{1}{\gamma }$ days, after which the development of clinical signs and onset of infectiousness are assumed to occur simultaneously (${I}_{C}^{S}$). Symptomatic infected cattle can then either die from the infection (with a probability *p*_*D*_) after a delay of $\frac{1}{\alpha }$ days completed in ${I}_{C}^{pd}$ [20], or recover with a probability (*1 − p*_*D*_) after a delay $\frac{1}{{\tau}_{CS}}$ days completed in ${I}_{C}^{pr}$. Thus, the duration of the latency period is the same for both the symptomatic and asymptomatic route, but the duration of the infectious periods is different [8]. Due to the short time frame of the simulations (< 1 year), recovered cattle are assumed to remain fully immune to new infections in the *R*_*C*_ compartment [10, 21]. Non–disease-related demographic processes as well as animal movements are ignored.

The vector population is subdivided into two compartments: the uncontaminated *S*_*V*_ and contaminated *I*_*V*_ compartments, as* Stomoxys* flies act as mechanical vectors [16]. Contaminated infective vectors remain capable of mechanically transmitting the virus for a period of $\frac{1}{{\tau}_{V}}$ days following exposure, after which they become susceptible again. The population of vectors is considered to be at a stable equilibrium, with equal birth and death rates *μ*.

The force of infection exerted on a susceptible cow is given by

$$\lambda_{C} { = }k p_{C} \frac{{I_{V} }}{{N_{C} }}$$

with *k* being the biting rate (day^−1^) of the vectors, *p*_*C*_ being the probability that a cow becomes infected upon a bite by a contaminated vector and *N*_*C*_ being the total number of cows.

The force of infection exerted on a susceptible vector is given by

$${\lambda}_{V}\mathrm{=}k \, \left({p}_{VS}\frac{{I}_{C}^{S}\mathrm{+}{I}_{C}^{pd}\mathrm{+}{I}_{C}^{pr}}{{N}_{C}}\mathrm{+}{p}_{VA}\frac{{I}_{C}^{A}}{{N}_{C}}\right)$$

with *p*_*VS*_ and *p*_*VA*_ being the probabilities that a susceptible vector becomes contaminated if it bites a symptomatic infectious cow and an asymptomatic infectious cow, respectively.

### Intervention strategies

Selective culling is assumed to start 3 days after the appearance of clinical signs in the first symptomatic animal (${I}_{C}^{S}$>0) to account for potential delays in suspicion notification, laboratory confirmation and the regulatory and logistical organisation of the culling. As part of a sensitivity analysis, delays of 5 and 7 days are also tested. All animals present are tested every second day after the initiation of the selective culling protocol, and positive individuals are culled (Figure 1B), assuming that diagnostic test results and the culling of positive animals are instantaneous. Diagnostic tests are assumed to be perfect when applied to symptomatic infectious cows, with P(detection|${I}_{C}^{S}$) = P(detection|${I}_{C}^{pr}$) = P(detection|${I}_{C}^{pd}$)= *Se*_*1*_= 1. However, because viraemia in asymptomatic infectious cows has been reported to be intermittent, diagnostic tests are assumed to yield a positive result with probability P(detection|${I}_{C}^{A}$)=*Se*_*2*_ on these cows, with *Se*_*2*_≤1. Diagnostic tests are further assumed to be able to detect infected cows during the second stage of their latent period, that is, before they become infectious, with a sensitivity P(detection|${E}_{C2}^{S}$) =* rSe*_*1*_ for the individuals that will become symptomatic and with the same sensitivity as the infectious stage for the individuals that will become infectious asymptomatic (P(detection|${E}_{C2}^{A}$) = *Se*_*2*_). The specificity of the test is assumed to be 1.

The different selective culling scenarios were defined based on a combination of: (1) the sensitivity of the diagnostic test in asymptomatic infectious cows (*Se*_*2*_​) and (2) time at which pre-infectious cows becomes detectable (parameter *a* in Figure 1). The test sensitivity in asymptomatic infectious cows was varied from 0 to 1 in increments of 0.1. The time at which pre-infectious cows become detectable was varied from 1 day (infected cows can begin testing positive the day after infection) to 7 days (infected cows can only test positive once they become infectious), assuming that both symptomatic and asymptomatic cows become infectious on an average of 7.3 days after infection [11]. To represent the time at which pre-infectious cows become detectable, the latent stage was subdivided into two stages. The first latent stage (${E}_{C1}$) represents pre-infectious cows that cannot yet be detected, while the second latent stage (${E}_{C2}^{A}$ and ${E}_{C2}^{S}$) represents pre-infectious cows that can be detected by diagnostic testing. Given an assumed total latent period of 7.3 days [11], the duration of the first latent stage corresponds to the delay until detectability, and the duration of the second latent stage was calculated to be 7.3 days minus this delay.

Finally, upon the initiation of selective culling (i.e. 3 days after the appearance of the first symptomatic animal), the model assumes that vector control is implemented such that the vector population is reduced instantaneously and maintained at this reduced level until the end of virus circulation in the herd. To account for different levels of effectiveness of insecticide treatment, due to varying degrees of vector resistance or protocol compliance, three levels of reduction in the vector population were considered: 0% (no vector control), 50% (moderately effective vector control) and 80% (effective vector control).

Parameter values are derived from field and experimental peer-reviewed studies and are synthesised in Table 1. The R_0_ of the model is derived using the Next Generation Matrix, provided in the Additional file 1 [22]. Sensitivity and elasticity analyses show that parameters with the largest relative effect on R_0_ are the biting rate and the vector-to-host ratio, which we set to the lower end of the reported values, indicating a best-case scenario (Additional file 1).

**Table 1:** **Parameter values used in the model**

Parameter	Definition	Value	References
**Infection dynamics parameters**			
1/μ	Lifespan of* Stomoxys*	12.5 days	[11]
1/τ_V_	Duration infectiousness of* Stomoxys* biting flies	5.5 days	[8, 11]
p_S_	Probability of showing clinical signs in cattle	0.5	[8]
1/τ_CS_	Duration of infectiousness of symptomatic cattle, for animals not dying from the infection	40 days	[8]
1/τ_CA_	Duration of infectiousness of asymptomatic cattle	15 days	[8]
1/α	Delay between symptom onset and death in cattle	20 days	[4]
1/b	Delay before subdivision into pre-death or pre-recovery in cattle	1 day	Assumed
p_D_	Probability of death for symptomatic cattle	0.12	[20]
1/a	Time at which a pre-infectious cows become detectable	1–7 days	Scenario range
δ	Duration of latency in cattle	7.3 days	[11]
1/γ	Duration between onset of detectability and onset of infectiousness	δ−1/a	Scenario range
k	Biting rate* Stomoxys*	3 days^−1^	[8, 18]
p_C_	Probability of cattle infection from being bitten by a contaminated* Stomoxys*	0.05	[8]
p_VS_	Probability of* Stomoxys* contamination from biting symptomatic infectious cattle	0.22	[8]
p_VA_	Probability of* Stomoxys* contamination from biting asymptomatic infectious cattle	0.006	[8]
**Parameters related to intervention strategies**			
r	Reduction of the test sensitivity for cattle in the second stage of their latent period compared with the infectious, symptomatic stage	0.5	Assumed
Se_1_	Test sensitivity for infectious symptomatic cattle	1	[6]
Se_2_	Test sensitivity for infectious asymptomatic cattle	0–1	Scenario range

### Simulation implementation

Intrinsic stochasticity arising from random variations in event timing was incorporated into the model using the Gillespie algorithm, following exponential distributions for the inter-event waiting times [23]. Additional analyses show the behaviour of the stochastic model for different values of R_0_ (Additional file 1). Simulations were run using the *SimInf* package in R 4.5.0 [24, 25]. For each intervention strategy, characterised by test sensitivity in asymptomatic infectious cows, time at which pre-infectious cows become detectable and reduction in vector population size following insecticide treatment, 1000 stochastic model simulations were performed. Each simulation started with fully susceptible cattle and vector populations, except for one cow in the first stage of the latent period. The model was run without intervention until a symptomatic animal appeared (detection time). For simulations leading to an outbreak (i.e. not going extinct directly after introduction), the state of all compartments at detection time was then used as the initial condition for the model with interventions, with each intervention defined as an “event” in the *SimInf* package. At the end of each simulation, we recorded the cumulative number of infected cattle (detected and undetected) and the cumulative number of infection episodes in vectors, the time to virus extinction (i.e. elimination or recovery of the last infected cattle) and the cumulative number of culled animals. For each outcome, the mean across 1000 stochastic repetitions is reported, together with the 5^th^ and 95^th^ percentiles (simulation interval).

## Results

**Figure 2:**
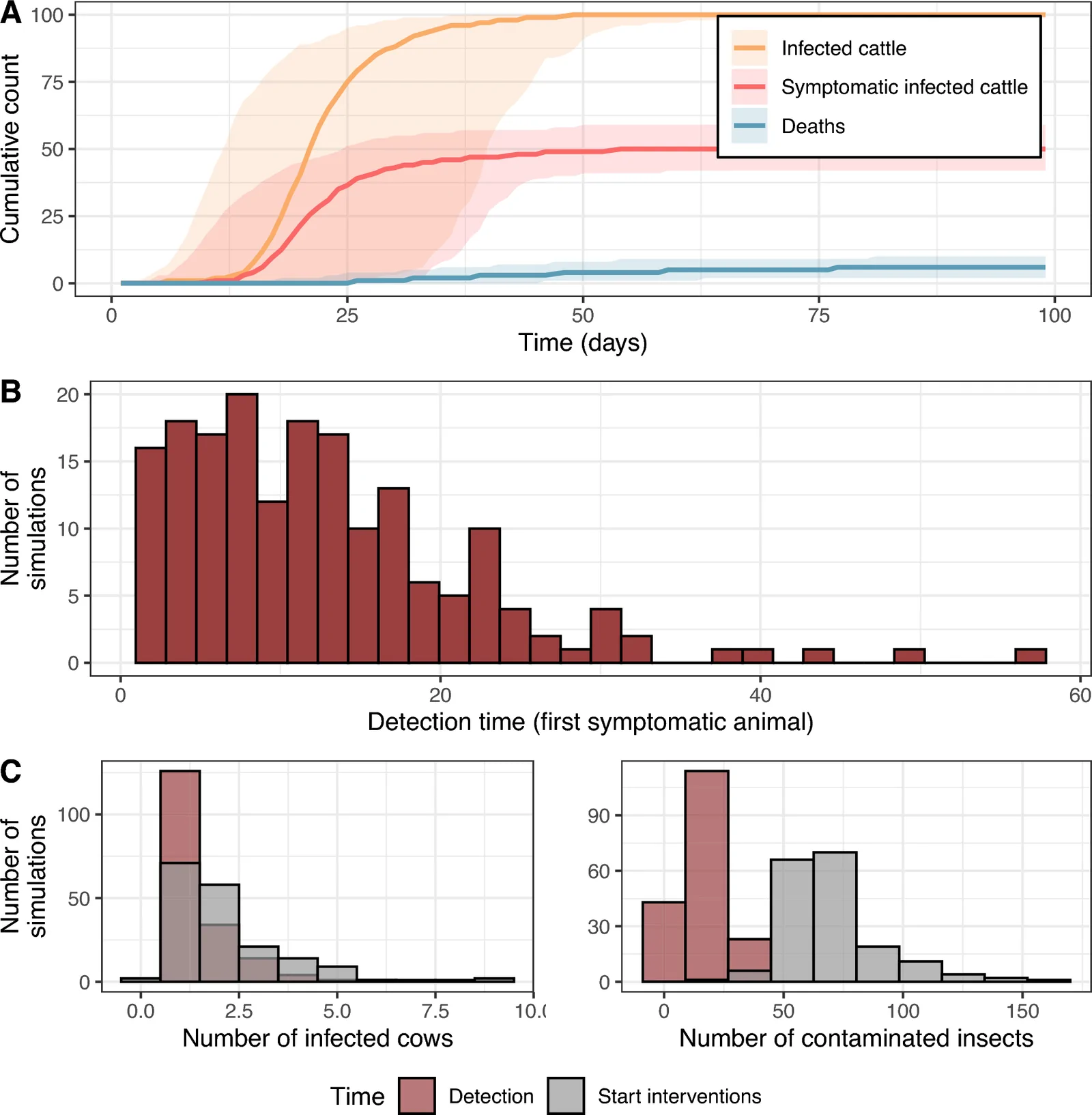
**Result of 1000 stochastic simulations of the model without interventions**. **A** Evolution of the cumulative infections in cattle, total, symptomatic and deaths over time. Solid lines are the median and the respective shaded areas are the 5^th^ and 95^th^ percentiles. **B** Distribution of the delay until the first symptomatic cow appears following virus introduction into the herd. **C** State of the system at detection time and at the start of interventions (3 days later).

The proposed model was first used to simulate LSD virus spread in the herd without intervention, with the aim to assess model validity against real-world contexts in which within-herd disease propagation was observed over longer time periods than in France due to the absence of infected herd depopulation [26]. The model exhibited an R_0_ of 19, consistent with previous model estimates [11, 27]. Without interventions, 91% of the simulations led to an outbreak following introduction. For the introductions resulting in outbreaks, the median final number of infected cattle was 100 (simulation interval: 100, 100), with 50 (simulation interval: 40, 59) symptomatic cattle. The delay in the appearance of the first symptomatic animal varied widely across simulations, with an average of 11 days (simulation interval: 1, 38) (Figure 2B). This variability is likely due to whether the first infected animal ultimately becomes symptomatic or remains asymptomatic. In the latter case, the first symptomatic individual would appear at least in the second generation of infected cows, leading to a higher infection prevalence in the herd at the time when the first symptomatic cow appears (Figure 2C). The incidence in cattle (Figure 2A) was compared with the only field observation reported in the literature, a farm where the only intervention was the culling of clinical cattle, in which approximately 40% of the herd eventually showed clinical signs [26].

### Effect of selective culling without vector control

**Figure 3:**
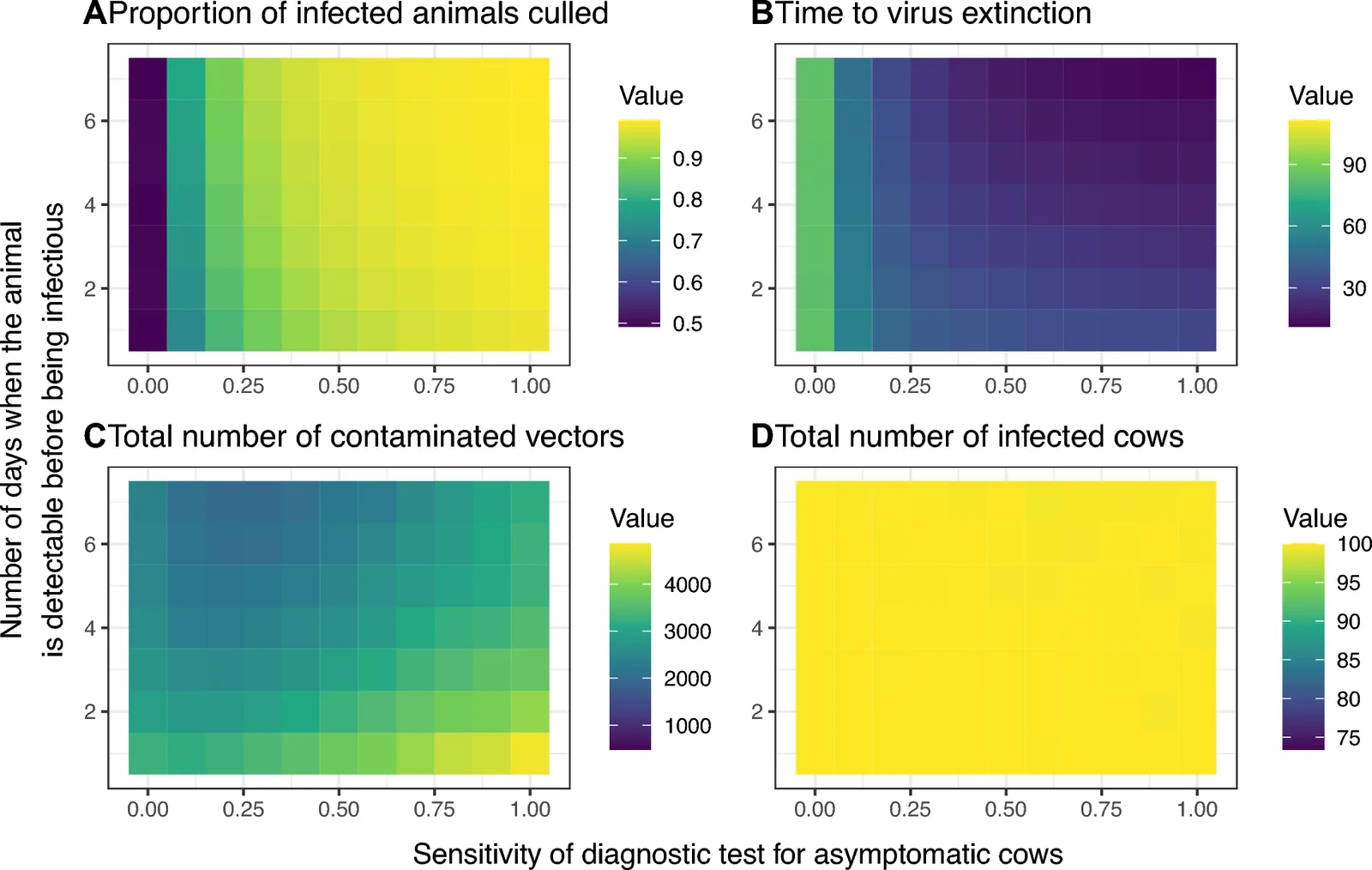
**Performance of the selective culling strategy without vector control**. For each combination of diagnostic test sensitivity in asymptomatic animals and time at which pre-infectious cows become detectable, each metric is calculated as the average across 1000 stochastic repetitions. These results assume no vector population reduction, and the implementation of a test-and-cull strategy implemented every 2 days from 3 days after the first clinical animal is detected. **A** Proportion of culled animals. **B** Time to virus extinction, calculated as the time required for the last infected animal to recover or die from the infection. **C** Total number of contaminated vectors. **D** Total number of infected animals.

Figure 3 shows the average of four simulation outcomes for various combinations of diagnostic test sensitivity in asymptomatic cattle and the time at which pre-infectious cows become detectable, with the start of interventions 3 days after detection of the outbreak. As expected, when the test is unable to detect asymptomatic individuals (first coloured column on the left in each panel), the proportion of infected animals culled corresponds to the proportion of symptomatic cattle (approx. 50%) and the average time to virus extinction is the longest (82 days). Also, the whole herd eventually becomes infected regardless of the test sensitivity and precocity. When animals can only be detected when they become infectious (first coloured row at the bottom of each panel), the number of contaminated vectors increases when the sensitivity of the test increases because symptomatic cows, which are more infectious, represent the larger proportion of the infected animals.

### Effect of a selective culling strategy combined with vector control

When the test-and-cull strategy is combined with vector control, overall epidemic metrics slightly improve. However, a relative reduction of the vector population of 50% (insecticide efficacy of 0.5) is insufficient, as for high levels of diagnostic test sensitivity in asymptomatic individuals (>0.5) and for timely detection of pre-infectious cows (at least 5 days prior to the onset of infectiousness), between 97 and 100 cows become infected. Similarly, epidemic metrics improve with a faster time to the start of intervention (Additional file 2).

**Figure 4:**
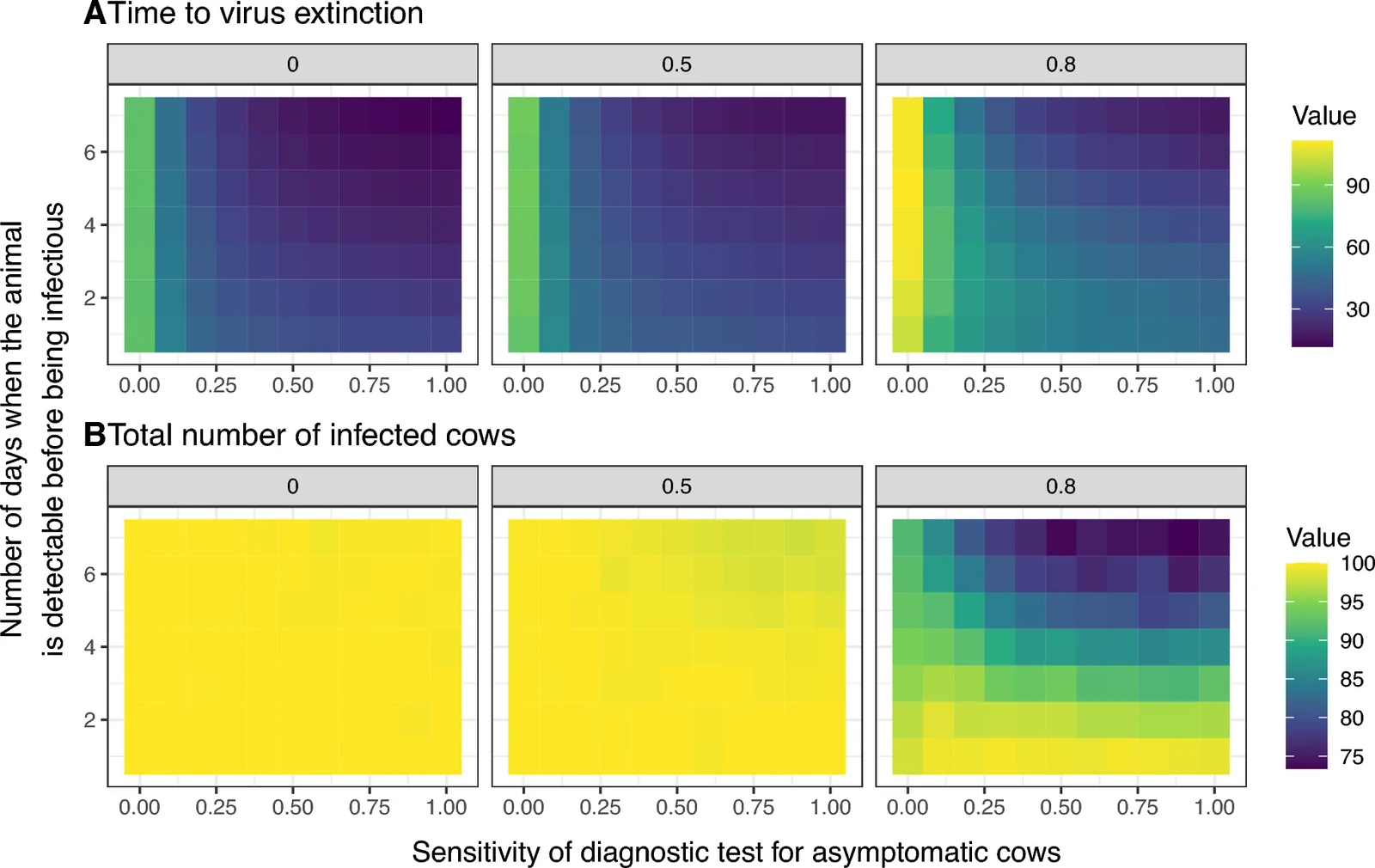
**Performance of the selective culling strategy for different efficacies of vector population control (0%, 50% and 80% in population reduction)**. For each combination of diagnostic test specificity and precocity and insecticide efficacy, the metric is calculated as the average across 1000 stochastic repetitions. These results assume a vector population reduction of 0%, 50% and 80%, respectively, from the moment the first clinical animal is detected until the end of the simulations, and the implementation of a test-and-cull strategy every 2 days from 3 days after the first clinical animal is detected. **A** Time to virus extinction, calculated as the time when the last infected animal recovers or dies from the infection. **B** Total number of infected animals.

Under the best-case scenario where vector population is reduced by 80%, epidemic metrics improve, illustrating more efficient outbreak management. Overall, for high levels of diagnostic test sensitivity in asymptomatic individuals (> 0.5) and timely detection of pre-infectious cows (at least 5 days prior to the onset of infectiousness), the average time to virus extinction is expected to be less than 35 days (simulation interval: 12, 61), and the average cumulative number of infected animals to be less than 80 (simulation interval: 29, 100). However, even when the sensitivity of the diagnostic test for asymptomatic animals is perfect and animals can be detected as soon as they become infected, it takes 18 days (simulation interval: 2, 28) between outbreak detection and extinction, with on average 75 cows (simulation interval: 26, 100) eventually becoming infected (Figure 4).

## Discussion

Using a within-herd, mechanistic model of LSD transmission, we have compared the efficacy of a selective culling strategy with potential diagnostic tests for asymptomatic individuals to total depopulation of the herd. The model reproduces within-herd transmission dynamics considering vectorial transmission is by* Stomoxys* biting flies. In the absence of any control measure, LSD spreads within the whole herd and persists for 150 days, exhibiting a high reproduction number consistent with previously published estimates [11, 26, 27]. The simulations show that even when the test-and-cull interventions are implemented every other day, starting 3 days after detection of the first symptomatic cow, together with highly effective vector population control and perfect detection of asymptomatic cows immediately upon infection, on average 75% of the herd becomes infected and the virus persists for 18 days in the herd. These epidemic metrics deteriorate when the time at which pre-infectious cows become detectable and sensitivity of the diagnostic test decreases. Current diagnostic tests of asymptomatic individuals are based on reverse transcription (RT)-PCR performed on blood samples, and are expected to have moderate performance due to transient and low-level viremia in early and subclinical infections [6, 28, 29]. Considering the PCR tests on blood samples of asymptomatic cows to have sensitivities of around 30% and 3 days of precocity, which is an optimistic scenario, the average time to virus extinction is expected to be approximately 60 days, with an average cumulative number of infected animals of around 94; this represents only a marginal within-herd benefit as compared to a full depopulation strategy. Additionally, as we assumed that animals are tested and culled every second day, such conditions would require 30 trips to the farm to perform the tests and cull the detected infected animals, resulting in a logistically intensive strategy compared to mass culling of the animals in the farm. The limited efficacy of the test-and-cull strategy is partly due to the late detection of infected herds, resulting in a high prevalence upon detection (Figure 2). Pre-emptive testing of herds at increased risk, for example, a herd neighbouring an infected herd, could reduce this detection delay and improve the effectiveness of the test-and-cull strategy (Additional file 2). However, this would substantially increase logistical demands, requiring sufficient staff and resources to test large numbers of animals in non-clinical herds.

Similarly, epidemic metrics worsen as insecticide efficacy decreases. For tests allowing perfect detection of asymptomatic individuals directly upon detection of infection, the total number of infected cows goes from 75 with 80% insecticide efficacy, to an average of 98 and 99 infected cows with 50% and 0% efficacy, respectively. Given that insecticide products report variable efficacies due to high levels of resistance to deltamethrine, the main insecticide product, it is difficult to estimate how to realistically calibrate this intervention [30, 31]. The model assumed an instantaneous decrease in the vector population, which later stabilised at that reduced level. In practice, the cattle would need to be treated at least every week to maintain the vector population at that reduced level [32]. Given the logistical constraints of such interventions as well as the important prevalence of the resistance of* Stomoxys* biting flies to insecticide products, we can assume an insecticide efficacy of 50% at best, resulting in marginal benefit of the selective culling strategy compared with a full depopulation.

There are a number of limitations. The estimations are limited by the model assumptions and parameter values. Due to the short time frame of the simulations, the model assumed a stable vector population regardless of temperature, precipitation or seasonality, which may not be realistic over longer periods. *Stomoxys calcitrans* was considered to be the only vector; however, other arthropods, such as *Aedes aegypti* or *Culicoides nubecolusus*, can mechanically transmit the virus with a reduced R_0_ [11]. Also, parameter values are subject to uncertainty. For example, contradictory transmission probabilities for symptomatic and asymptomatic cows have been reported in the literature [8, 11], and the vector-to-host ratio varies widely depending on farming practices [33, 34]. Similarly, the mean duration of infectiousness in contaminated* Stomoxys* flies was assumed to be 5.5 days, based on a controlled infection experiment in which flies fed on an infectious calf were tested by quantitatve (q)PCR at regular intervals [9]. However, under field conditions,* Stomoxys* flies are likely to experience more rapid decontamination of the mouthparts through successive feeding attempts. Moreover, qPCR positivity does not necessarily indicate infectivity, as viral DNA may be present at concentrations too low to cause transmission. Consequently, the duration of infectiousness under natural conditions could be shorter than that estimated under experimental conditions. Overall, in the current model, we used parameter values at the lower end of the reported ranges, reflecting a best-case scenario, and conducted additional sensitivity analyses (Additional file 1). Finally, the model assumed an exponential distribution of the time spent in each compartment, which may affect the timing of the different interventions, but the overall results of the management strategy performance metrics were robust to Erlang-distributed durations in the compartments (Additional file 4).

The aim of controlling a notifiable disease in a herd is to prevent pathogen spread to other farms and to limit the consequences of the disease from a collective perspective. The model presented here is limited to within-herd transmission and does not take into account between-farm transmission. The fast transmission dynamics led to a high prevalence in the herd, resulting in most cattle becoming infected. Although only 50% of the infected animals display clinical signs, subclinical animals also contribute to the transmission dynamics, potentially threatening neighbouring herds [8, 11]. Additionally, a longer time to virus extinction and a higher prevalence of both infected cows and* Stomoxys* biting flies reflect a higher infectious potential of the herd and would result in an infection pressure on other farms. How that pressure is exerted is likely highly context-dependent (season, spatial setting, animal movements and farm connectivity) and should be evaluated in further work. To further assess the impact of selective culling, the modelling approach presented here will be embedded within a between-farm framework, allowing evaluation of the consequences of a selective strategy at a territorial scale.

Finally, this study primarily examines management strategies centred on test-and-cull approaches in the absence of vaccination. A vaccine is proposed against LSD [35], and French herds in the affected regions underwent emergency vaccination during the epidemic. The current work aims at presenting the model and comparing depopulation strategies to test-and-cull strategies for different diagnostic test regimes, focusing on the initial epidemic context with unvaccinated livestock. In such a context, vaccination would occur, at best, at the start of the intervention period (3 days after the detection of the first case, which occurs 2 to 30 days after the outbreak begins; Figure 2), with effective protection only developing around 21 days after vaccination [35]. This implies that herd-level protection would be reached approximately 25–33 days after the onset of infection. According to Figure 2, this would be at a stage at which between 75% and almost 100% of the animals would already be infected, which would limit the benefit of vaccination. Future work will extend the comparison of the two culling strategies in herds that had already been vaccinated to inform future management strategies in such contexts.

## Conclusion

In this paper, we present a within-herd transmission model of LSD that captures key biological features of the disease and incorporates a flexible test-and-remove module to simulate selective culling under varying diagnostic test performance. This model can inform the highly debated management of LSD in France by providing farm-level epidemiological and economic predictions under a range of scenarios, including vaccination.

## Supplementary Information


**Additional file 1: Model behaviour analysis.****Additional file 2: Effect of intervention timing.****Additional file 3: Effect of the herd size.****Additional file 4: Effect of an Erlang distribution of the duration in cattle infectious and latent stages.**

## Data Availability

This manuscript does not contain any data. The code used to run the analyses and make the figures is available on [https://github.com/cla-delec/LSD_intrafarm_model].
