# Supplementary material for: Selective culling and the potential persistence of lumpy skin disease in cattle herds: insights from a mechanistic transmission model
Source: Vet Res. 2026 Aug 29;57:160. doi: 10.1186/s13567-026-01828-5 (PMC13526299; doi:10.1186/s13567-026-01828-5)
Supplement: Supplementary file 1 [file 13567_2026_1828_MOESM1_ESM.pdf]

Additional file 1: Model behaviour analysis

Belonging to the manuscript "*Selective culling and the potential persistence of lumpy skin disease in cattle herds: insights from a mechanistic transmission model*"

Clara Delecroix, Gaël Beaunée, Stéphane Bertagnoli, Thibaut Lurier, Brandon Hayes, Sébastien Picault, Guillaume Fournié, Timothée Vergne

Sensitivity and elasticity analysis

To evaluate the effect of parameter values on R_0, we performed a sensitivity and elasticity analysis (Matser et al., 2009). The sensitivity and elasticity of R_0 to a given parameter indicate how a change in that parameter affects R_0, with elasticity offering the advantage of being a relative measure. Elasticity values can be compared when the parameters have different scales. The sensitivities and elasticities are computed numerically using the package *popbio* in R (Stubben & Milligan, 2007), with the values provided in Table 1. The next generation matrix (NGM) K_L of the model without intervention is used to calculate the numerical value of R_0. The NGM is computed following (Diekmann et al., 2010) as K_L=-$T\ \Sigma^{-1}$, with

$$T = \begin{pmatrix} 0 & 0 & 0 & 0 & 0 & 0 & 0 & kp_C \\ 0 & 0 & 0 & 0 & 0 & 0 & 0 & 0 \\ 0 & 0 & 0 & 0 & 0 & 0 & 0 & 0 \\ 0 & 0 & 0 & 0 & 0 & 0 & 0 & 0 \\ 0 & 0 & 0 & 0 & 0 & 0 & 0 & 0 \\ 0 & 0 & 0 & 0 & 0 & 0 & 0 & 0 \\ 0 & 0 & 0 & 0 & 0 & 0 & 0 & 0 \\ 0 & 0 & 0 & kp_{VS}\frac{N_V}{N_C} & kp_{VS}\frac{N_V}{N_C} & kp_{VS}\frac{N_V}{N_C} & kp_{VA}\frac{N_V}{N_C} & 0 \end{pmatrix}$$

And

$$\Sigma = \begin{pmatrix} -a & 0 & 0 & 0 & 0 & 0 & 0 & 0 \\ ap_S & -\gamma & 0 & 0 & 0 & 0 & 0 & 0 \\ a(1-p_S) & 0 & -\gamma & 0 & 0 & 0 & 0 & 0 \\ 0 & \gamma & 0 & -b & 0 & 0 & 0 & 0 \\ 0 & 0 & \gamma & 0 & -\tau_{CA} & 0 & 0 & 0 \\ 0 & 0 & 0 & bp_D & 0 & -\alpha & 0 & 0 \\ 0 & 0 & 0 & b(1-p_D) & 0 & 0 & -\tau_{CS} & 0 \\ 0 & 0 & 0 & 0 & 0 & 0 & 0 & -\tau_V-\mu \end{pmatrix}$$

The elasticity and sensitivity analyses show that the biting rate *k*, the probability of infection for cattle upon a bite p_C, and the vector-to-host ratio (N_V/N_C) have the largest effect on R_0 (without intervention) (Figure S1). We chose parameter values in a best-case scenario, with a biting rate and vector-to-host ratio in the low range of reported estimates (Sanz-Bernardo et al., 2021; Vergne et al., 2021). Thus, the transmission dynamics are likely underestimated.

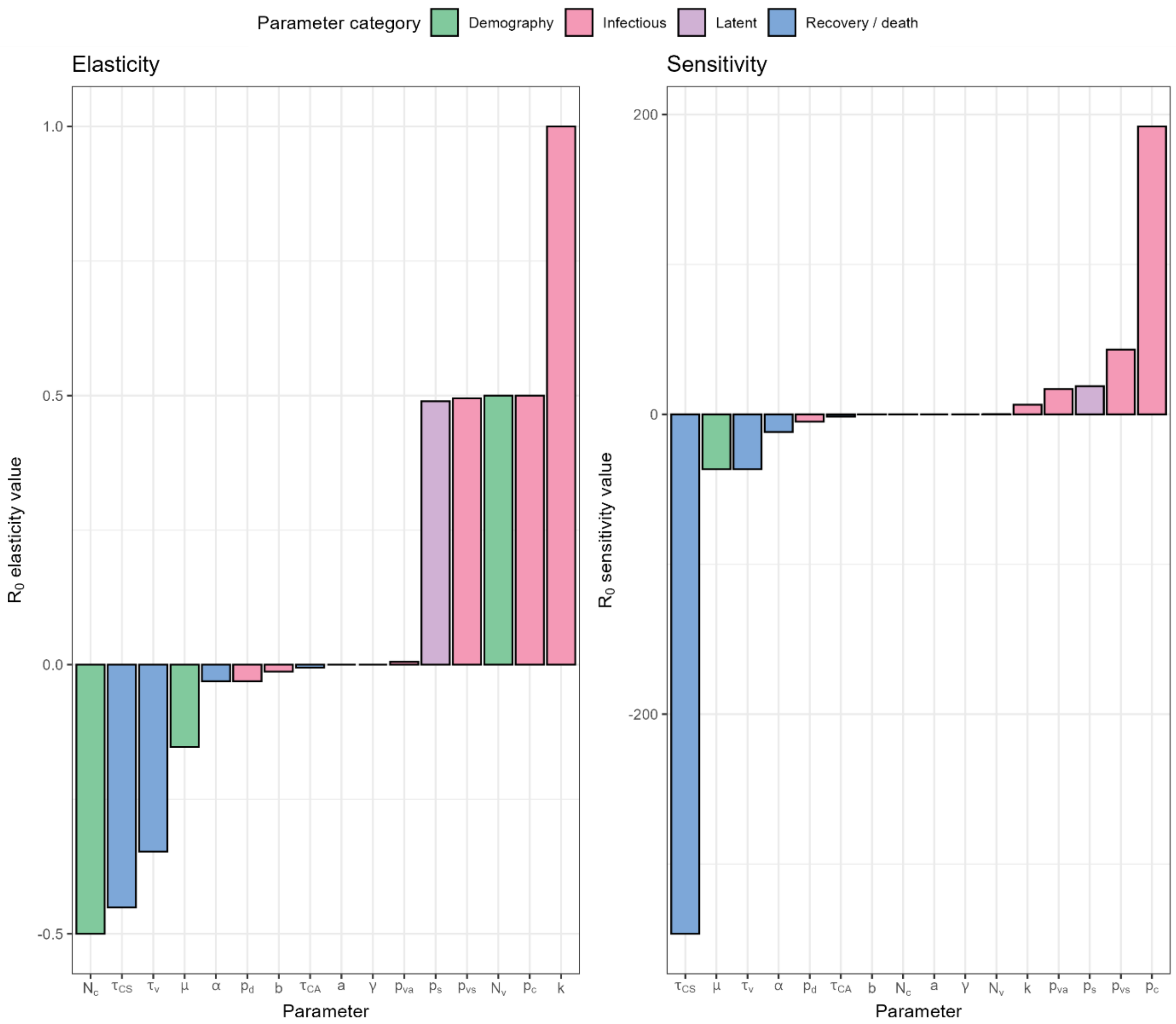

Figure S1: Elasticity and sensitivity analysis of the model without interventions, for all the model parameters reported in Table 1.

Model analysis

To assess model consistency, we performed stochastic simulations for different values of R_0. We use the biting rate *k* as the control parameter and vary it from 0 to 3, corresponding to R_0 values ranging from 0 to 19. The model undergoes a bifurcation at $R_0=1$ ($k=0.15$), going from a disease-free stable equilibrium to an endemic equilibrium. For each value of R_0, we run 1000 stochastic simulations of the model without interventions and calculate the proportion of simulations resulting in an extinction of the epidemic (defined as a total number of infected cows below five), the total number of infected cows and the total number of infected Stomoxys biting flies (Figure S2). Consistent with the analysis of the model, few infected cows and Stomoxys biting flies are observed for R_0 below 1, and the majority of simulations result in an extinction (>90%). When R_0 crosses the critical threshold of 1, the number of infected cows and Stomoxys biting flies increase and the proportion of extinction decreases.

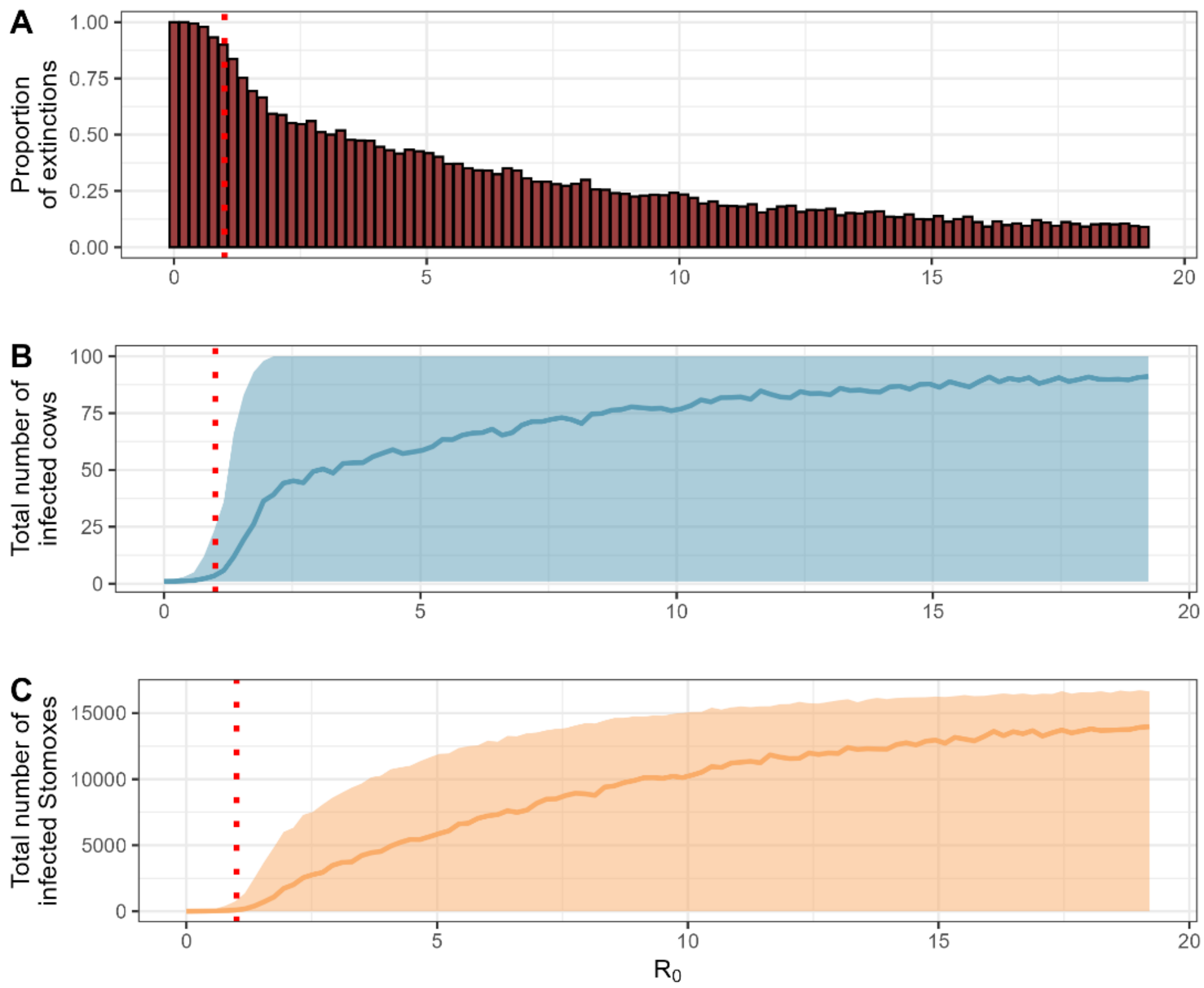

Figure S2: Behaviour of 1000 stochastic simulations for different values of R_0. The red dotted line indicates the critical threshold of R_0=1. A Proportion of simulations resulting in extinctions, defined as <5 infected cows. B Total number of infected cows, average (solid line) over 1000 simulations and 95% simulation interval (shaded area) for each value of R_0. C Total number of infected Stomoxys biting flies, average (solid line) over 1000 simulations and 95% interval (shaded area) for each value of R_0.
