# Supplementary material for: Selective culling and the potential persistence of lumpy skin disease in cattle herds: insights from a mechanistic transmission model
Source: Vet Res. 2026 Aug 29;57:160. doi: 10.1186/s13567-026-01828-5 (PMC13526299; doi:10.1186/s13567-026-01828-5)
Supplement: Supplementary file 2 [file 13567_2026_1828_MOESM2_ESM.pdf]

Additional file 2: Effect of intervention timing

Belonging to the manuscript "*Selective culling and the potential persistence of lumpy skin disease in cattle herds: insights from a mechanistic transmission model*"

Clara Delecroix, Gaël Beaunée, Stéphane Bertagnoli, Thibaut Lurier, Brandon Hayes, Sébastien Picault, Guillaume Fournié, Timothée Vergne

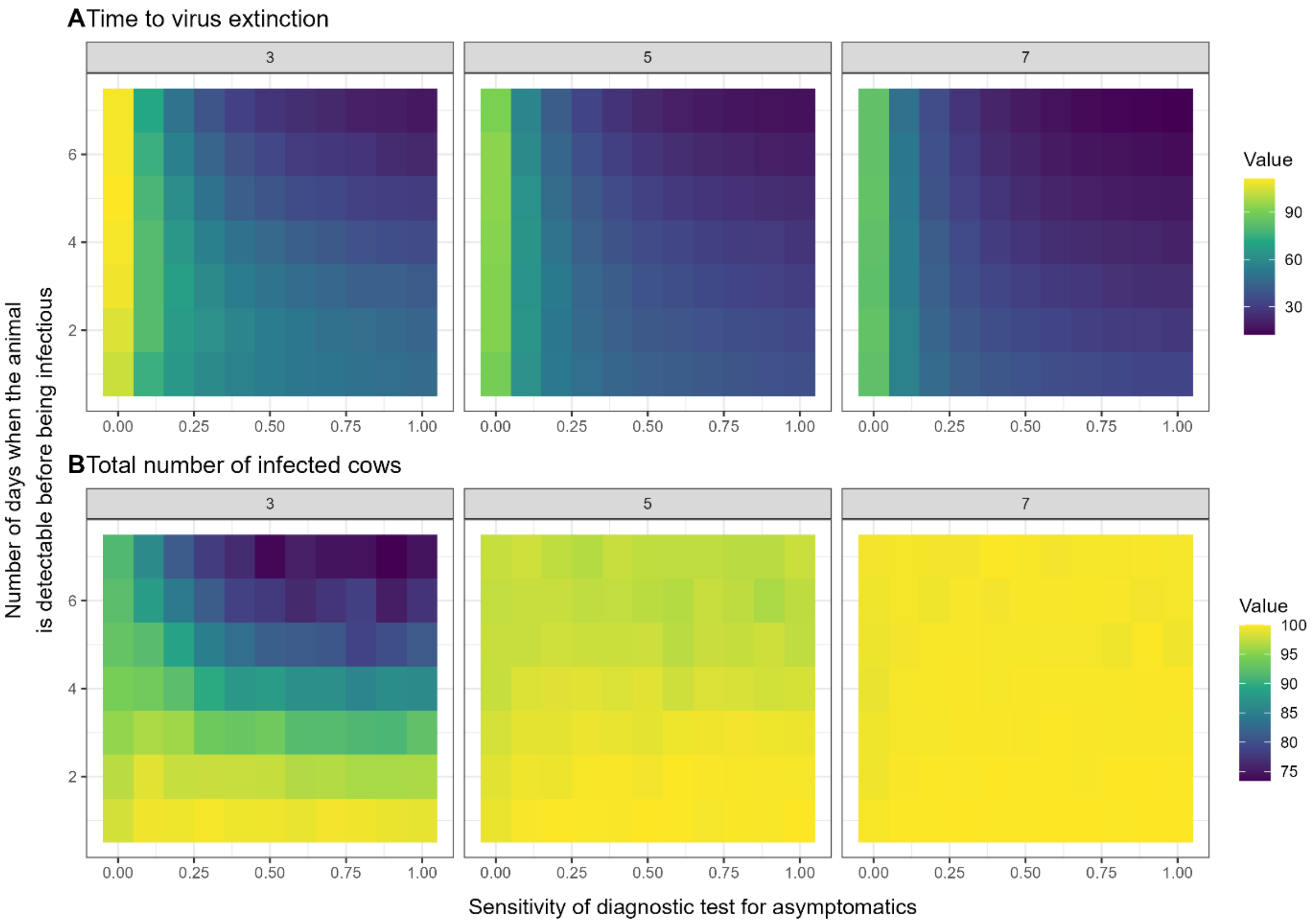

Figure S3: Performance of the selective culling strategy for different timing of start of interventions (3, 5 and 7 days post detection). For each combination of diagnostic test specificity and precocity and start of interventions, the metric is calculated as the average across 1000 stochastic repetitions. These results assume that interventions start respectively 3, 5 and 7 days after the detection of the first clinical cow in the herd, and are implemented as a test-and-cull strategy implemented every two days. A Time to virus extinction, calculated as the time when the last infected animal recovers or dies from the infection. B Total number of infected animals.
