# Supplementary material for: Selective culling and the potential persistence of lumpy skin disease in cattle herds: insights from a mechanistic transmission model
Source: Vet Res. 2026 Aug 29;57:160. doi: 10.1186/s13567-026-01828-5 (PMC13526299; doi:10.1186/s13567-026-01828-5)
Supplement: Supplementary file 3 [file 13567_2026_1828_MOESM3_ESM.pdf]

Additional file 3: Effect of the herd size

Belonging to the manuscript "*Selective culling and the potential persistence of lumpy skin disease in cattle herds: insights from a mechanistic transmission model*"

Clara Delecroix, Gaël Beaunée, Stéphane Bertagnoli, Thibaut Lurier, Brandon Hayes, Sébastien Picault, Guillaume Fournié, Timothée Vergne

We assessed the effect of herd size using herds of 50, 100 (main text) and 150 cows. For these three values, R_0 is respectively 27, 19 and 16.

Overall, the results remain qualitatively unchanged (Figure S4).

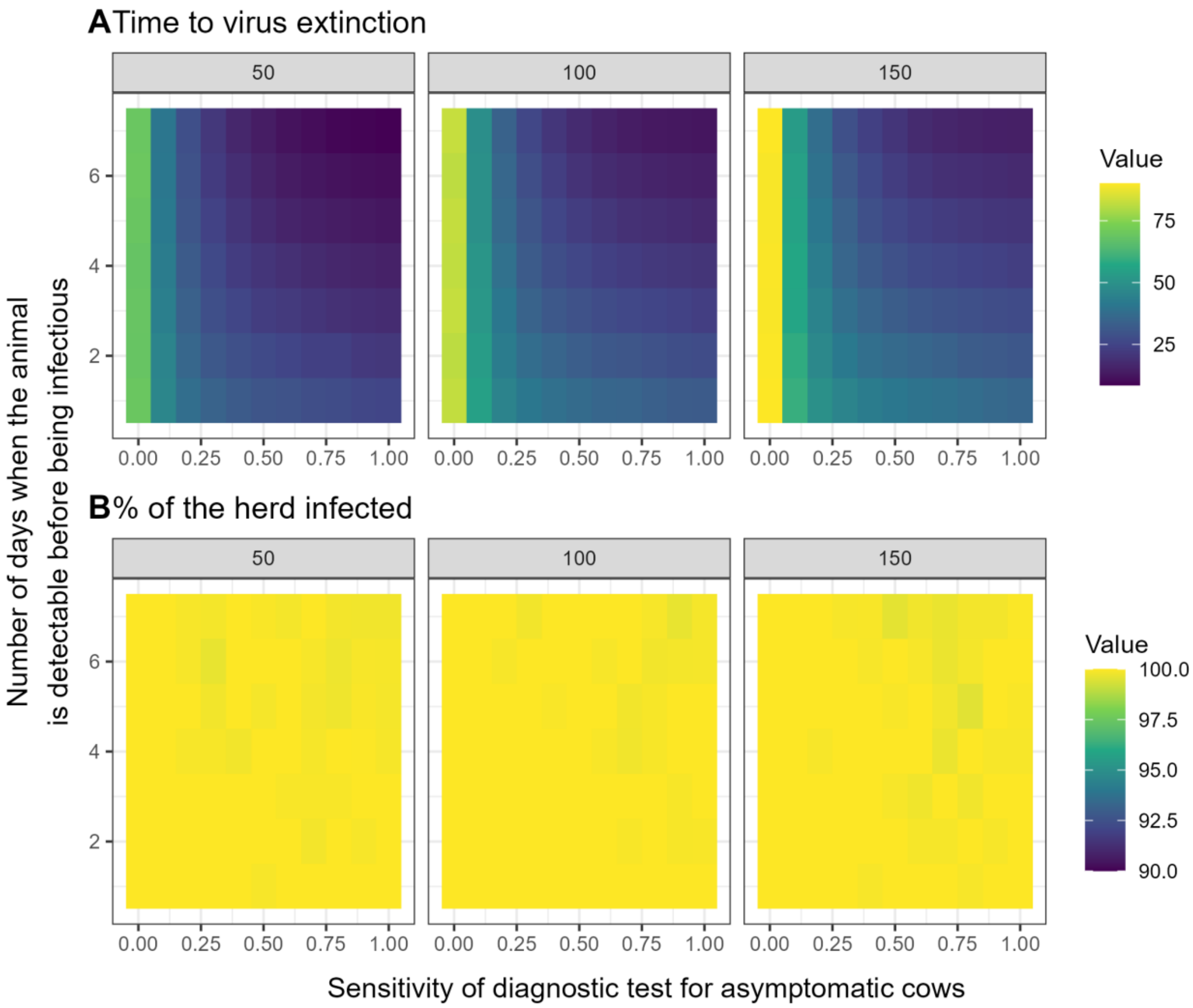

Figure S4: Performance of the selective culling strategy for different herd sizes (50, 100 and 150 cows). For each combination of diagnostic test specificity and precocity and start of interventions, the metric is calculated as the average across 1000 stochastic repetitions. These results assume that interventions start 3 days after the detection of the first clinical cow in the herd, and are implemented as a test-and-cull strategy implemented every two days. A Time to virus extinction, calculated as the time when the last infected animal recovers or dies from the infection. B Percentage of the herd infected.
