# Supplementary material for: Selective culling and the potential persistence of lumpy skin disease in cattle herds: insights from a mechanistic transmission model
Source: Vet Res. 2026 Aug 29;57:160. doi: 10.1186/s13567-026-01828-5 (PMC13526299; doi:10.1186/s13567-026-01828-5)
Supplement: Supplementary file 4 [file 13567_2026_1828_MOESM4_ESM.pdf]

Additional file 4: Effect of an Erlang distribution of the duration in cattle infectious and latent stages

Belonging to the manuscript "*Selective culling and the potential persistence of lumpy skin disease in cattle herds: insights from a mechanistic transmission model*"

Clara Delecroix, Gaël Beaunée, Stéphane Bertagnoli, Thibaut Lurier, Brandon Hayes, Sébastien Picault, Guillaume Fournié, Timothée Vergne

An Erlang distribution was tested by splitting the compartments into j compartments. We picked j=4 as a trade-off between having sufficient compartments to distinguish from the exponential distribution and a reasonable computational time. All the compartments related to latency and infectiousness (E_{C1}, E_{C2}^{A}, E_{C2}^{S}, I_{C}^{A}, I_{C}^{pd}, and I_{C}^{pr}) were split. For instance, E_{C1} is split into E_{C1_1} E_{C1_2} E_{C1_3} and E_{C1_4}. The compartment I_{C}^{S} was not split as it is an artificial compartment used to divide symptomatic individuals into the ones that will die from the infection and the ones that will recover without affecting the duration spent in the compartment.

Overall, the results remain qualitatively unchanged (Figure S5). The main difference is a reduced number of extreme values in the detection time (maximum of 61 days for the exponentially distributed model against 26 for the Erlang-modified model) (Figure S6B). Consistently, the prevalence in the herd and in Stomoxys biting flies is reduced (Figure S6C). However, the performance metrics remain comparable to the exponential model.

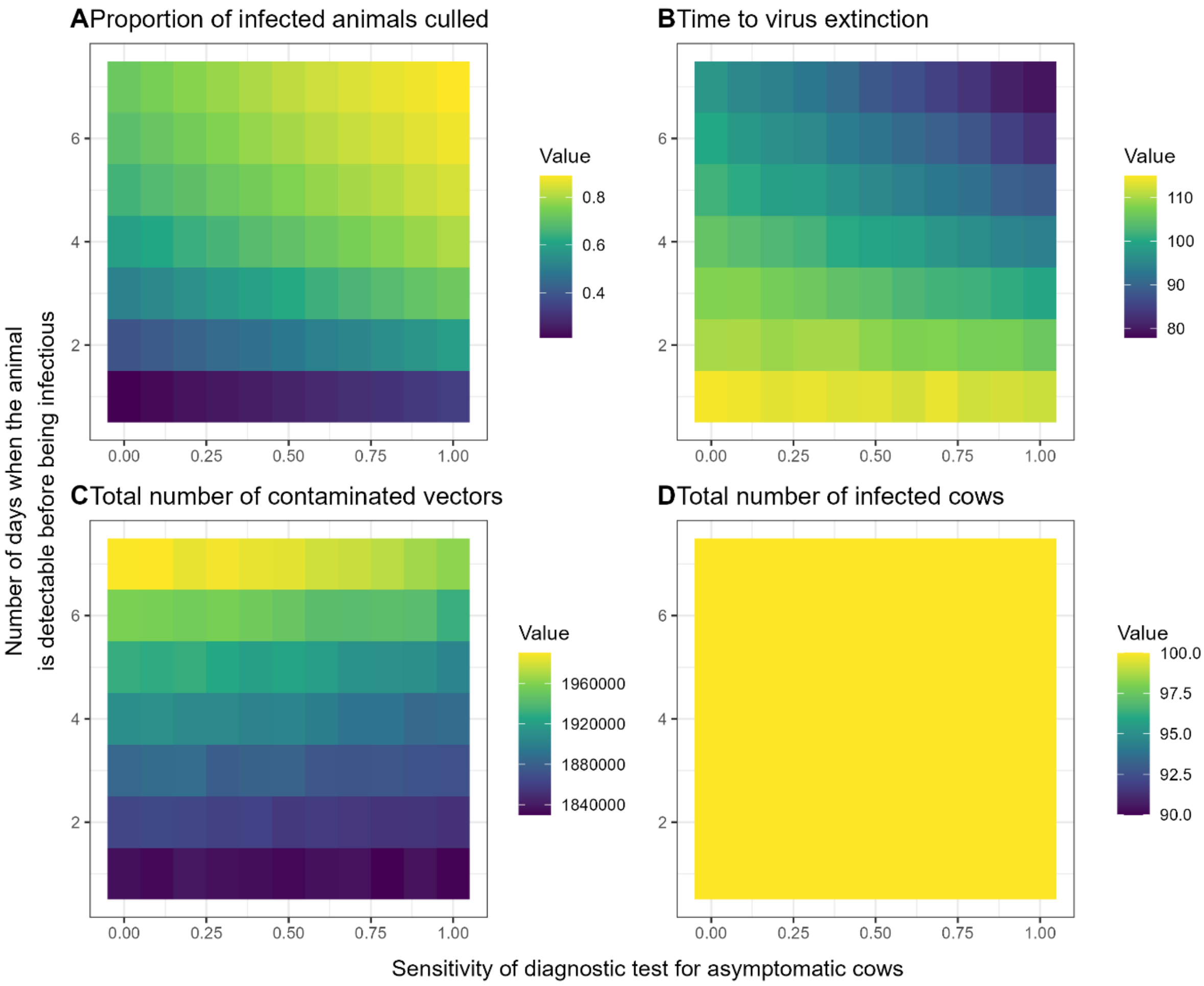

Figure S5: Performance of the selective culling strategy for a modified model with Erlang compartments. For each combination of diagnostic test sensitivity in asymptomatic animals and time at which pre-infectious cows become detectable, each metric is calculated as the average across 1000 stochastic repetitions. These results assume a test-and-cull strategy implemented every two days from three days after the first clinical animal is detected, and no vector population control. A: Number of culled animals; B: Time to virus extinction, calculated as the time required for the last infected animal to recover or die from the infection; C: Total number of contaminated vectors; D: Total number of infected animals.

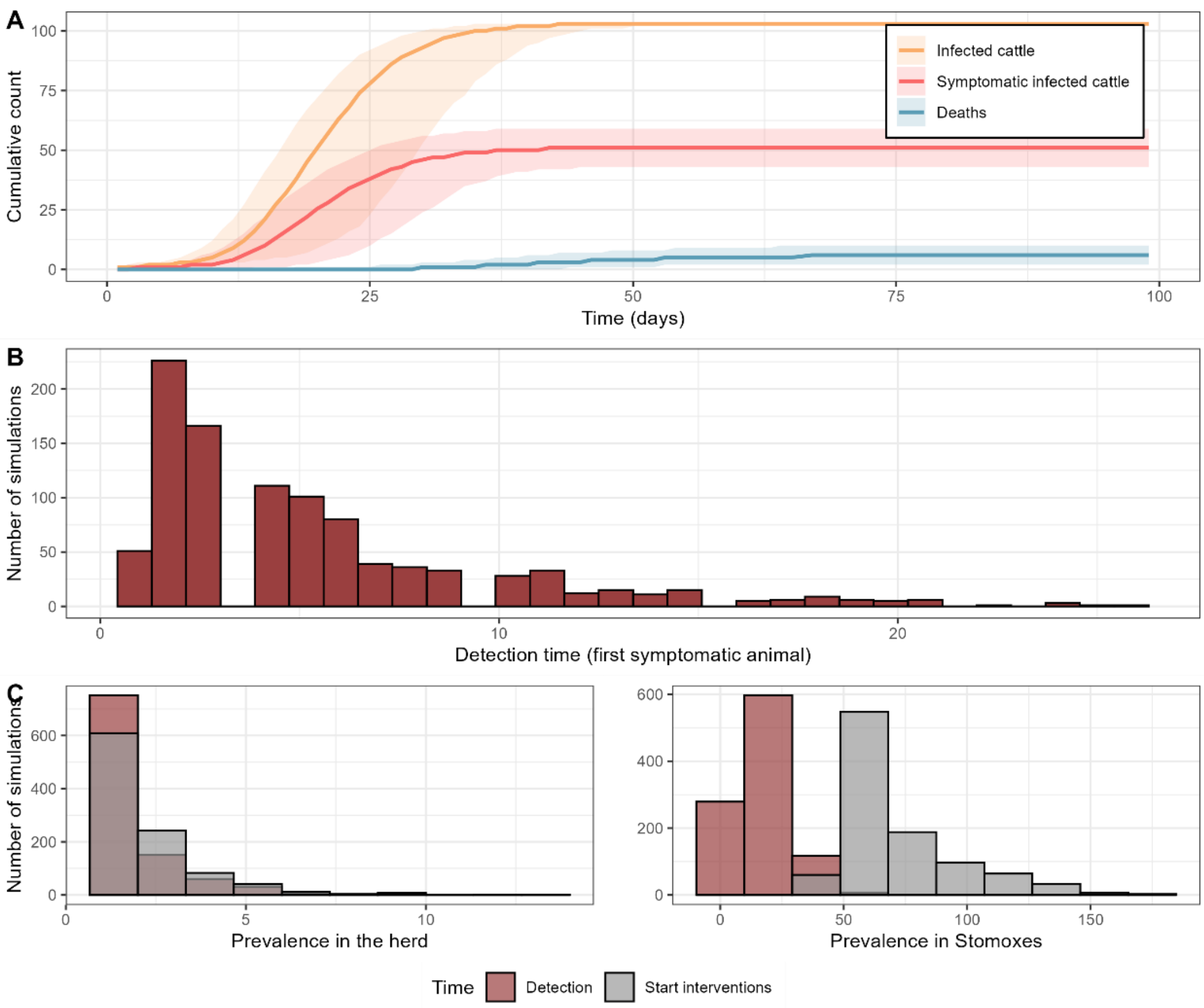

Figure S6: Result of 1000 stochastic simulations of the model without interventions, for a modified model with Erlang compartments. A: Evolution of the cumulative infections in cattle, total, symptomatic and deaths over time (median, 5th and 95th percentiles). B: Distribution of the delay until the first symptomatic cow appears following virus introduction into the herd. C: State of the system at detection time, and at the start of interventions (7 days later). The prevalence is shown as the number of infected cows and flies, respectively.
